# Cerebral Blood Flow Is Reduced in Severe Myalgic Encephalomyelitis/Chronic Fatigue Syndrome Patients During Mild Orthostatic Stress Testing: An Exploratory Study at 20 Degrees of Head-Up Tilt Testing

**DOI:** 10.3390/healthcare8020169

**Published:** 2020-06-13

**Authors:** C (Linda) M.C. van Campen, Peter C. Rowe, Frans C. Visser

**Affiliations:** 1 Stichting CardioZorg, 2132 HN Hoofddorp, The Netherlands; fransvisser@stichtingcardiozorg.nl; 2 Department of Paediatrics, John Hopkins University School of Medicine, Baltimore, MD 21205, USA; prowe@jhmi.edu

**Keywords:** orthostatic intolerance, cerebral blood flow, 20 degree tilt table testing, myalgic encephalomyelitis, chronic fatigue syndrome, postural orthostatic tachycardia syndrome, stroke volume index, cardiac index

## Abstract

Introduction: In a study of 429 adults with myalgic encephalomyelitis/chronic fatigue syndrome (ME/CFS), we demonstrated that 86% had symptoms of orthostatic intolerance in daily life. Using extracranial Doppler measurements of the internal carotid and vertebral arteries during a 30-min head-up tilt to 70 degrees, 90% had an abnormal reduction in cerebral blood flow (CBF). A standard head-up tilt test of this duration might not be tolerated by the most severely affected bed-ridden ME/CFS patients. This study examined whether a shorter 15-min test at a lower 20 degree tilt angle would be sufficient to provoke reductions in cerebral blood flow in severe ME/CFS patients. Methods and results: Nineteen severe ME/CFS patients with orthostatic intolerance complaints in daily life were studied: 18 females. The mean (SD) age was 35(14) years, body surface area (BSA) was 1.8(0.2) m^2^ and BMI was 24.0(5.4) kg/m^2^. The median disease duration was 14 (IQR 5–18) years. Heart rate increased, and stroke volume index and end-tidal CO_2_ decreased significantly during the test (*p* ranging from <0.001 to <0.0001). The cardiac index decreased by 26(7)%: *p* < 0.0001. CBF decreased from 617(72) to 452(63) mL/min, a 27(5)% decline. All 19 severely affected ME/CFS patients met the criteria for an abnormal CBF reduction. Conclusions: Using a less demanding 20 degree tilt test for 15 min in severe ME/CFS patients resulted in a mean CBF decline of 27%. This is comparable to the mean 26% decline previously noted in less severely affected patients studied during a 30-min 70 degree head-up tilt. These observations have implications for the evaluation and treatment of severely affected individuals with ME/CFS.

## 1. Introduction

Myalgic encephalomyelitis/chronic fatigue syndrome (ME/CFS) patients have a high prevalence of orthostatic intolerance [1,2]. In a study of 429 adult ME/CFS patients, we recently demonstrated that 86% had orthostatic intolerance symptoms during daily life. Moreover, during a 30-min head-up tilt table test, 90% had an abnormal cerebral blood flow (CBF) reduction as assessed by extracranial Doppler measurements [2,3]. This abnormal CBF reduction was not only present in ME/CFS patients with well-defined heart rate and blood pressure abnormalities during tilt testing, like orthostatic hypotension, postural orthostatic tachycardia syndrome (POTS) and syncope [4,5,6], but also in ME/CFS patients with a normal heart rate and blood pressure response to upright posture [2]. The mean CBF reduction of 26% in the entire study population with ME/CFS was significantly different from the 7% reduction observed in healthy controls in response to the same orthostatic stress.

Documenting an abnormal cerebral blood flow is helpful in guiding therapy for orthostatic intolerance (OI). As described in the IOM report: “Orthostatic intolerance is defined as a clinical condition in which symptoms worsen upon assuming and maintaining upright posture and are ameliorated (although not necessarily abolished) by recumbency” [1]. Symptoms of orthostatic intolerance sought in the history of patients “are those caused primarily by [1] cerebral underperfusion (such as light-headedness, near-syncope or syncope, impaired concentration, headaches, and dimming or blurring of vision), or [2] sympathetic nervous system activation (such as forceful beating of the heart, palpitations, tremulousness, and chest pain. Other common signs and symptoms of orthostatic intolerance are fatigue, a feeling of weakness, intolerance of low-impact exercise, nausea, abdominal pain, facial pallor, nervousness, and shortness of breath”.

A limitation of the extracranial Doppler measurements is that image acquisition lasts between 2 and 7 min [2]. In our recent study, patients were excluded if they were unable to maintain the upright position during the acquisition period, and also if a rapid drop in heart rate and blood pressure prevented complete image acquisition. Moreover, patients can develop post-exertional malaise after conventional 60–90 degree head-up orthostatic stress testing [1]. Due to these problems—image acquisition time, the potential for post-exertional malaise and inability to stand long enough— conventional orthostatic testing may not be advisable in severe ME/CFS patients. Moreover, in previous work, we showed that 15 of 444 patients could not complete standing during the head-up tilt test [2]. Wyller et al. described a different method of orthostatic stress testing [7], which involved a low-grade head-up tilt test of 20 degrees in 27 ME/CFS adolescents. The rationale was that a 70 degree head-up tilt testing is associated with a high rate of false-positive results in adolescents. Using the 20 degree tilt over a period of 15 min, the authors showed that heart rate, blood pressure and stroke volume index changes were different in ME/CFS adolescents when compared with age- and gender-matched controls.

Assuming that severe ME/CFS patients cannot tolerate prolonged standing during tilt testing and may have more hemodynamic abnormalities including a rapid decline of blood pressure, the aim of the current study was to test the hypothesis that reduced cerebral blood flow and reduced stroke volume index/cardiac index could also be confirmed in severe ME/CFS patients during 15 min of low-grade (20 degree) head-up tilt testing.

## 2. Materials and Methods

### 2.1. Patients

From June 2019 to April 2020, 139 patients visited the outpatient clinic of the Stichting CardioZorg, Hoofddorp, the Netherlands, because of a suspicion of ME/CFS. This cardiology clinic specializes in diagnosing and treating adults with ME/CFS. All patients were evaluated by the same clinician (FVC). During the first visit, it was determined whether patients satisfied the criteria for CFS and ME, taking the exclusion criteria into account. Patients were classified as having CFS, chronic fatigue or no chronic fatigue as defined by Fukuda and colleagues [8] and as having ME or no ME as defined by Carruthers and colleagues [9]. Disease severity was scored by a clinician according to the ICC, with severity ranging between mild, moderate, severe and very severe. This was classified according to the paper as: “Symptom severity impact must result in a 50% or greater reduction in a patient’s premorbid activity level for a diagnosis of ME. Mild: approximately 50% reduction in activity, moderate: mostly housebound, severe: mostly bedbound and very severe: bedbound and dependent on help for physical functions” [9]. The clinician ascertained for the presence of orthostatic intolerance symptoms in daily life like dizziness/light-headedness, prior (near)-syncope and nausea, among others, as well as triggering events like standing in a line. Over this 10 month period, 137 patients met the criteria for ME/CFS, 19 (14%) of whom met the criteria for severe ME/CFS. In those patients, an orthostatic stress test was performed at a low-grade head-up tilt angle of 20 degrees. The study was carried out in accordance with the Declaration of Helsinki. All ME/CFS patients gave informed, written consent. The study was approved by the medical ethics committee of the Slotervaart Hospital, Amsterdam, The Netherlands (reference number P1736).

### 2.2. Head-Up Tilt Test with Cerebral Blood Flow and Stroke Volume Measurements

Measurements were performed as described previously [3], with the main exception being that patients were positioned supine for 20 min before being tilted head-up to 20 degrees for a maximum of 15 min instead of the more classic approach of 70 degrees for 25–30 min. They were investigated in the morning, at least 3 h after a light breakfast or in the afternoon 3 h after a light lunch. No formal hydration protocol was applied, but subjects were asked to ingest an ample amount of fluid. If patients developed severe orthostatic symptoms, the test was stopped prematurely, but after upright image acquisition. The test was prematurely stopped in 6 patients due to an increase in symptoms. Heart rate and systolic and diastolic blood pressures were continuously recorded by finger plethysmography using the Nexfin device (BMeye, Amsterdam, The Netherlands). Heart rate and blood pressures were extracted from the device and imported into an Excel spreadsheet. End-tidal PCO_2_ (PetCO_2_) was monitored using a Lifesense device (Nonin Medical, Minneapolis, MN, USA).

### 2.3. Cerebral Blood Flow Determination by Doppler Echographic Measurements

Internal carotid artery and vertebral artery Doppler flow velocity frames were acquired by one operator (FCV), using a Vivid-I system (GE Healthcare, Hoevelaken, the Netherlands) equipped with a 6–13 MHz linear transducer. Flow data of the internal carotid artery (ICA) on the right and on the left side were obtained ~1.0–1.5 cm distal to the carotid bifurcation and of the vertebral artery (VA) on the right and on the left side, data were obtained at the C3–C5 level. Care was taken to ensure the insonation angle was less than 60 degrees, that the sample volume was positioned in the center of the vessel and that it covered the width of the vessel. High-resolution B mode images, color Doppler images and the Doppler velocity spectrum (pulsed wave mode) were recorded in one frame. The order of imaging was fixed: left internal carotid artery (ICA), left vertebral artery (VA), right internal carotid artery (ICA) and right vertebral artery (VA). At least two consecutive series of six frames per artery were recorded. The recording time intervals of the first and last imaged artery were noted and these times were corrected to the times of a radio clock, setting the start of tilt at 0 min. Heart rate and blood pressures of the echo recording time intervals were averaged. In the supine position, image acquisition started 8 (2) min prior to tilting (supine data) and during the upright position acquisition started at 10 (4) min. Based on data from healthy controls during a 30-min 70 degree head-up tilt, we defined an abnormal reduction in CBF as a >13% decline during the tilt compared to the supine values [2]. Analysis is described in the data-analysis section.

### 2.4. Stroke Volume Determination by Doppler Echocardiographic Measurements:

To determine stroke volume, velocity time integral (VTI) frames were obtained in the resting supine position and the upright position as previously described [10]. The aortic VTI was measured using a continuous wave Doppler pencil probe (GE P2D: 2 MHz) connected to a Vivid I machine (GE, Hoevelaken, NL, USA) with the transducer positioned in the suprasternal notch. A maximal Doppler signal was assumed to be the optimal flow alignment. At least 2 frames of 6 s were obtained. Echo Doppler recordings were stored digitally. From a transthoracic echocardiogram, the diameter of the left ventricular outflow tract (LVOT) was obtained. Analysis is described in the data-analysis section.

### 2.5. Data Analysis

The changes in heart rate and blood pressure during the head-up tilt test were classified according to the consensus statements [4,6]: normal heart rate and blood pressure response, classic orthostatic hypotension (a decrease of over 20 mmHg in systolic blood pressure and over 30 mmHg in the case of a systolic blood pressure over 140 mmHg, or a decrease of 10 mmHg in diastolic blood pressure from 1–3 min after tilt), delayed orthostatic hypotension (a decrease of over 20 mmHg in systolic blood pressure and over 30 mmHg in the case of a systolic blood pressure over 140 mmHg, or a decrease of 10 mmHg in diastolic blood pressure after 3 min post tilt), postural orthostatic tachycardia syndrome (POTS) (a sustained increase of at least 30 bpm within 10 min, without a significant decrease in BP) and syncope or near-syncope.

Blood flows of the internal carotid and vertebral arteries were calculated offline by an investigator (CMCvC) who was unaware of the patient severity status and unaware of the hemodynamic outcome of the head-up tilt test. Vessel diameters were manually traced by CMCvC on B-mode images, from the intima to the opposite intima. Surface area was calculated: the peak systolic and end diastolic diameters were measured, and the mean diameter was calculated as: mean diameter = (peak systolic diameter × 1/3) + (end diastolic diameter × 2/3) [11]. Blood flow in each vessel was calculated from the mean blood flow velocities times the vessel surface area and expressed in mL/min. Flow in the individual arteries was calculated in 3–6 cardiac cycles and data were averaged. Total cerebral blood flow was calculated by adding the flow of the four arteries. We previously demonstrated that this methodology had good intra- and inter-observer variability [3].

To determine stroke volumes, the velocity time integral was measured offline by manual tracing of at least 6 cardiac cycles, using the GE EchoPac post-processing software, by one operator (CMCvC). Stroke volumes were calculated from the corrected VTI and the LVOT cross-sectional area, as described previously [12,13]. Stroke volume index (SVI) was calculated by the equation: corrected LVOT cross-sectional area times the corrected aortic VTI, divided by the body surface area (BSA; DuBois formula) (mL/m^2^). SVIs of the separate cycles were averaged. Cardiac index was calculated by the equation: SVI × heart rate/1000 (L/min/m^2^).

### 2.6. Statistical Analysis

Data were analyzed using Graphpad Prism version 6.05 (Graphpad software, La Jolla, CA, USA). All continuous data were tested for normal distribution using the D’Agostino–Pearson omnibus normality test, and presented as mean (SD) or as median with the IQR, where appropriate. For continuous data, paired and non-paired *t*-tests were used for comparison, when appropriate. Linear regression analysis was performed correlating the percent cardiac index change with the percent cerebral blood flow change. A *p* value of <0.05 was considered significant.

## 3. Results

### Patient Clinical and Echo Doppler Data

Nineteen patients with a severe grade of ME/CFS were studied (18 females). Baseline characteristics were as follows: mean age 35 (14) years, height 171 (4) cm, weight 70 (17) kg, BSA 1.8 (0.2) m^2^ and BMI 24.0 (5.4) kg/m^2^. The median disease duration was 14 (IQR 5–18) years. All patients met the Fukuda criteria for CFS, and all met the ICC criteria for ME. Daily life orthostatic intolerance symptoms were reported by all 19 ME/CFS patients. At the time of the test, no patients were being treated with drugs influencing heart rate or blood pressure and none were being treated with selective serotonin reuptake inhibitors (SSRI). Of the 19 ME/CFS patients, four met the criteria for POTS at 20 degrees. None of the patients developed orthostatic hypotension or vasovagal syncope at 20 degrees.

Table 1 shows the tilt table results of the study participants during the low-grade 20 degree head-up tilt testing. Heart rate increased from 83 (13) supine to 104 (23) bpm (*p* < 0.0001), and end-tidal CO_2_ decreased from 38 (3) to 29 (6) mmHg (*p* < 0.001). Cerebral blood flow supine was 619 (68) mL and at the end of study was 453 (63) mL, a decline of 27 (5)%, with a decrease ranging between 21% and 37%. All 19 patients met criteria for an abnormal reduction in cerebral blood flow, being a more than 13% reduction. Stroke volume index decreased from 36 (6) to 25 (5) mL/m^2^, a decline of 31 (8)%. Cardiac index fell from 2.9 (0.5) to 2.1 (0.4) L/min/m^2^, a decline of 27 (7%).

**Figure 1:**
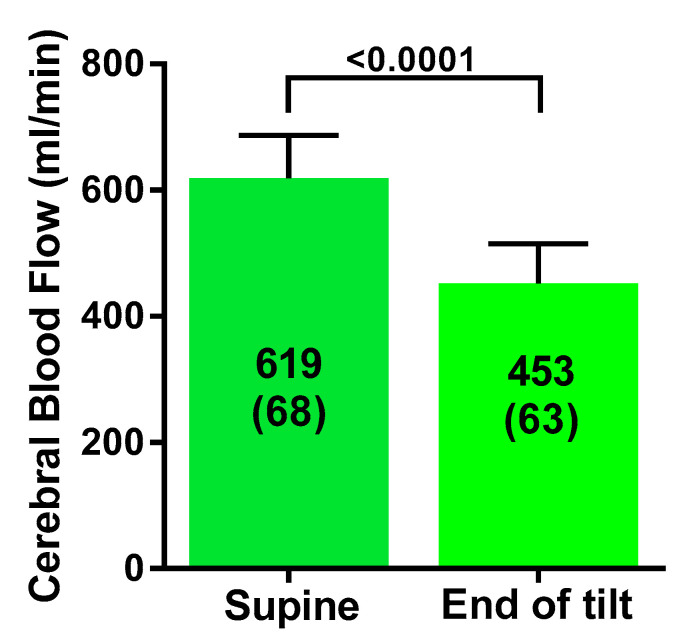
Cerebral blood flow in mL/min supine and at the end of a 20 degree tilt in severe ME/CFS patients.

**Figure 2:**
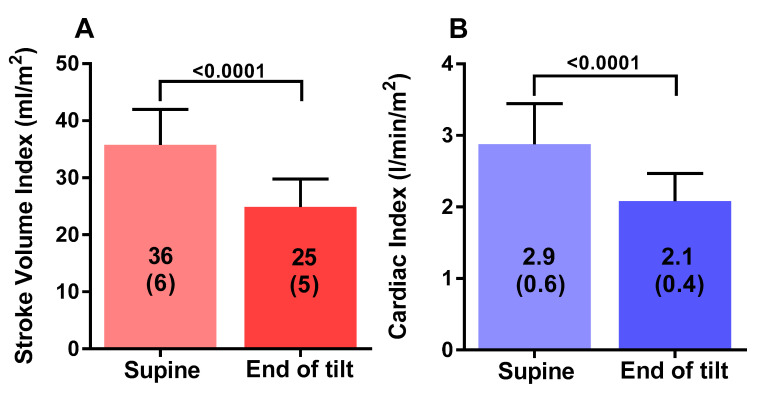
Stroke volume index in mL/m^2^ supine and at the end of a 20 degree tilt in severe ME/CFS patients (panel **A**). Cardiac index in L/min/m^2^ supine and at the end of a 20 degree tilt in severe ME/CFS patients (panel **B**).

**Figure 3:**
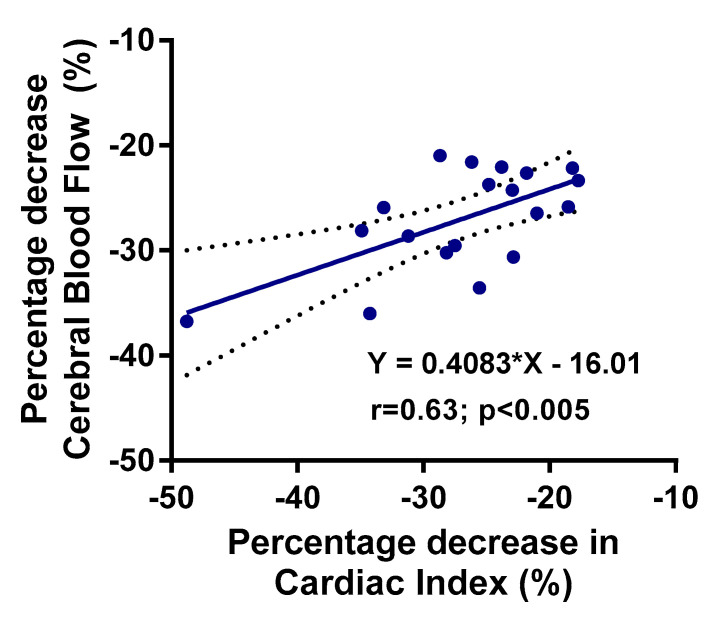
Correlation between the percent decrease in cardiac index and the percent decrease in cerebral blood flow.

Figure 1 shows the graphical representation of the supine cerebral blood flow and the cerebral blood flow at the end of the low-grade head-up tilt test: the difference was highly statistically significant (*p* < 0.0001). Figure 2 shows the hemodynamic changes in stroke volume index supine and at the end of the test in panel A, and the hemodynamic changes in cardiac index supine and at the end of the test in panel B. Both stroke volume index and cardiac index declined significantly (both *p* < 0.0001). Figure 3 shows the relation between the change in cardiac index and the change in cerebral blood flow: this relation was significant (*p* < 0.005).

Table 2 shows the comparison between ME/CFS patients with a normal heart rate and blood pressure response and those with POTS. The heart rate in POTS patients was significantly higher at the end of the study compared with the normal heart rate and blood pressure patients (*p* < 0.05). All other data were not significantly different between the two groups. Table 2 also shows the comparison per group of supine and end of study data. All values were statistically significantly different, except for diastolic blood pressure in the POTS group.

## 4. Discussion

The main finding of this exploratory study is that in severe ME/CFS patients, a significant reduction in cerebral blood flow can be provoked during a brief 20 degree head-up tilt test. The combination of low-grade head-up tilt testing and extracranial Doppler echography has not been described before. The 27% reduction in cerebral blood flow after 15 min compares to the 26% reduction observed after 30 min of 70 degree head-up tilt in a less severely affected population of ME/CFS patients [2]. We also observed a significant reduction in stroke volume index during this mild orthostatic stress, and found a significant correlation between the decrease in cardiac index and the decrease in cerebral blood flow. Finally, even with this low orthostatic stress, four patients fulfilled the heart rate criteria for POTS, while none of the patients had an orthostatic hypotension or (near) syncope. A milder orthostatic stress of 20 degrees also allowed the accurate measurement of CBF declines that might have been difficult to measure in those who have rapid drops in blood pressure associated with classical orthostatic hypotension or vasovagal syncope when tested at 70 degrees [2]. It remains to be determined whether a 20 degree tilt angle would be adequate for the diagnosis of orthostatic intolerance/significant cerebral blood flow reduction in less severely affected ME/CFS patients.

A previous study with 20 degree head-up tilt testing for 15 min in adolescent CFS patients showed a significantly higher heart rate and lower stroke volume index early after tilting (0.5–2.5 min after onset of tilt) in CFS patients compared with controls [7]. The present study in adults confirms the changes in stroke volume index during 20 degree tilt testing in CFS adolescents. Remarkably, supine heart rates were higher in our patient population (mean 35 years) than in the adolescent CFS population of Wyller et al. (mean 15 years) [7]. A large scale population study in healthy participants showed that resting heart rate in 15 year old adolescents normally is slightly higher than in adults over 20 years [14]. Our observation of a higher resting heart rate in the severe ME/CFS patient population might be related to disease severity, with a higher heart rate in more severe patients. However, this needs to be studied in a larger sample size.

It is generally assumed that part of the OI symptomatology is related to cerebral hypoperfusion [4,5,15,16]. One technique to study cerebral perfusion is transcranial Doppler. Using this technique, OH and POTS have been studied in different diseases and under different physiological conditions like aging, high-altitude, space flights and heat stress [17,18,19,20,21,22,23,24,25,26,27,28,29,30,31,32] However, it has been noted that OI symptoms during HUT may be present, even in the absence of abnormalities of heart rate or blood pressure [33,34,35,36]. Three recent studies used transcranial Doppler to investigate cerebral perfusion in patients with a normal HUT and without an abnormal HR and BP response like POTS or OH [33,34,36]. The three studies found that the blood flow velocity decrease in patients with a normal HUT but with OI symptoms was larger than in healthy volunteers and patients without OI, and similar to the patients with POTS or OH. These observations suggest that cerebral hypoperfusion is not only present in POTS and OH patients, but also in patients with OI symptoms without POTS and OH.

Several points about the study findings deserve emphasis. First, in our earlier study using a 70 degree tilt test, the decrease in stroke volume index was 31% at 15 min post tilt with no differences between mild, moderately and severely affected ME/CFS patients [10]. The stroke volume index decrease in the present study was 30%. This suggests that mild orthostatic stress testing in severe ME/CFS patients results in similar stroke volume index reductions when compared to 70 degree testing in ME/CFS patients of less severe disease. Further studies are needed to compare stroke volume changes at the different tilt angles in this patient population with varying degrees of disease severity.

Second, in a previous study, we found that cerebral blood flow reduction was 26% during a 70 degree tilt test [2]. In the present study, a cerebral blood flow decrease of 27% was observed. In contrast, studies using transcranial Doppler have shown no differences in cerebral blood flow velocities between CFS patients and healthy controls both at low-grade and high-grade tilt angles [37]. However, the authors showed that the end-tidal CO_2_ values were lower in CFS patients compared with controls, at all tilt angles. Previous studies have shown that hypocapnia can reduce intracranial vessel diameters, thereby altering the relation between flow velocity changes and hemodynamic changes [38,39,40]. Therefore, the absence of a difference in cerebral blood flow velocities of patients versus controls using transcranial Doppler may be related to end-tidal CO_2_-related vasoconstriction of the middle cerebral artery in CFS patients. Vasoconstriction leads to increases in cerebral flow velocities, resulting in non-significant differences in the TCD measurement between CFS patients and controls. The more direct measurement using extracranial Doppler identifies the global reduction in CBF that can be missed with transcranial Doppler. The similarity of cerebral blood flow reduction in the present study and our previous study despite the lower degree of orthostatic stress and the shorter tilt is most likely related to the more severe disease status of patients in the present study [2]. Consistent with our previous study [2], the results of this study again clearly demonstrate that reduced cerebral blood flow is a cardinal contributor to orthostatic intolerance symptoms in ME/CFS patients.

Third, as seen in our previous study and other recent studies using transcranial Doppler, we again demonstrated that an orthostatic intolerance/abnormal cerebral blood flow reduction may be present without heart rate and blood pressure abnormalities [2,33,34,35,36]. Patients with a normal heart rate and blood pressure response during a tilt test would have been misclassified as having no abnormalities. The present study suggests that cerebral blood flow measurements are needed in order to more accurately measure the prevalence of orthostatic intolerance in ME/CFS patients.

Fourth, the data of this study extend the observation that hypocapnia is significant in ME/CFS patients and is a likely contributor to reductions in cerebral blood flow [2,34,41]. The end-tidal CO_2_ reductions also support the observation that 20 degrees is sufficient to provoke similar cerebral blood flow changes in severe ME/CFS patients compared with a less severely diseased group of ME/CFS. When hypocapnia is observed, a focus on respiration depth and speed has the potential to be one of the therapeutic guidance options for these patients to lessen orthostatic intolerance complaints. Another possible factor besides vasculature, autonomic nervous system and end-tidal CO_2_ might be altered blood cell behavior, especially red blood cell [42,43].

Fifth, in a previous review on the relation between acute cardiac output changes and cerebral blood flow changes, Meng et al. found in healthy volunteers that a 30% reduction in cardiac output resulted in a 10% reduction in cerebral blood flow [44]. In the present study, we also found a significant relation between the reduction in cardiac index and cerebral blood flow. This provides further evidence for the validity of the extracranial Doppler measurements to determine cerebral blood flow. Furthermore, from the linear regression analysis in the present study, we calculated that a 30% reduction in cardiac index resulted in a 28% reduction in cerebral blood flow, which is much larger than the 10% in healthy volunteers in the review of Meng et al. [44]. Whether these differences are specific to ME/CFS are related to the use of transcranial Doppler vs. extracranial Doppler or are related to the significant reductions in end-tidal CO_2_ in the present study will need to be determined in future and larger studies.

Finally, even during a brief 20 degree tilt, four (21%) patients developed POTS. This has not been described before and might be a reflection of the severity of the disease. It also implies that the diagnosis of POTS cannot be dismissed when patients have complaints suggestive of POTS in non-standing positions like sitting or lying down with a slight head-up position.

### 4.1. Clinical Implications

Patients are advised to lie down when they experience orthostatic intolerance complaints. Our findings of a clinically significant cerebral blood flow reduction at just 20 degrees suggest that a slight head-up position may not be adequate enough to resolve symptoms of orthostatic intolerance in some patients. Furthermore, the European Society of Cardiology syncope guidelines and other papers advocate the use of a nocturnal head-up position of more than 10 degrees to prevent nocturnal polyuria and the consequent circulatory underfilling [45,46,47,48]. In light of the presented results, this advice has the potential to be detrimental in some ME/CFS patients.

### 4.2. Limitations

This study only included ME/CFS patients who were bedbound, and we caution that the 20 degree head-up tilt angle needs further study before it can replace longer 70 degree tilt angles for assessing less severely impaired ME/CFS patients. Comparisons of the hemodynamic and cerebral blood flow abnormalities of 20 and 70 degrees of tilting are needed. We also did not include healthy controls for comparison. It is possible that healthy controls would have little or no perturbation in response to a 20 degree head-up angle, which would have the effect of widening the physiologic differences between ME/CFS patients and controls. Whether disease severity differences lead to differences in cerebral blood flow reduction needs to be studied in the future. Finally, while it is reasonable to expect that the 20 degree abbreviated tilt test would be less taxing than a longer 70 degree tilt test, and therefore less likely to provoke post-exertional malaise, this hypothesis remains to be tested.

## 5. Conclusions

This study demonstrates that a short 15-min tilt using a mild 20 degree head-up angle is sufficient to provoke a clinically significant reduction in cerebral blood flow in patients with severe ME/CFS. This method of orthostatic testing has the potential to improve the assessment of the prevalence of orthostatic intolerance in severely affected ME/CFS patients who are reluctant to undergo a 70 degree tilt. In this patient population, a milder orthostatic stress was able to confirm the following: cerebral blood flow abnormalities in the absence of heart rate and blood pressure abnormalities; POTS is a small subset; and associated reductions in end-tidal CO_2_, stroke volume and cardiac index.

## Figures and Tables

**Table 1:** Tilt table test data of severe myalgic encephalomyelitis/chronic fatigue syndrome (ME/CFS) patients (n = 19).

	Supine	End of Study	*p*-Value
Heart rate (bpm)	83 (13)	104 (23)	<0.0001
Systolic blood pressure (mmHg)	134 (11)	138 (14)	0.10
Diastolic blood pressure (mmHg)	80 (9)	86 (9)	0.0006
End-tidal CO_2_ (mmHg)	38 (3)	29 (6)	<0.0001
Cerebral blood flow (mL/min)	619 (68)	453 (63)	<0.0001
Stroke volume index (mL/m^2^)	36 (6)	25 (5)	<0.0001
Cardiac index (L/min/m^2^)	2.9 (0.6)	2.1 (0.4)	<0.0001
Cerebral blood flow %change	−27 (5)%		
Stroke volume index %change	−31 (8)%		
Cardiac index %change	−27 (7)%		

%change: percent change from supine data.

**Table 2:** Tilt table test data of severe ME/CFS patients: normal heart rate and blood pressure response and postural orthostatic tachycardia syndrome (POTS) response at 20 degrees tilting.

	Group 1 Norm BPHR n = 15	Group 2 POTS n = 4	*p*-Value Group 1 vs. Group 2	*p*-value Group 1 Supine vs. End Tilt	*p*-Value Group 2 Supine vs. End Tilt
HR supine (bpm)	80 (12)	91 (10)	0.14		
HR end-tilt (bpm)	98 (21)	126 (10)	0.03	0.001	0.0002
SBP supine (mmHg)	135 (11)	132 (7)	0.63		
SBP end-tilt (mmHg)	141 (13)	126 (3)	0.06	0.02	0.19
DBP supine (mmHg)	80 (10)	80 (4)	0.98		
DBP end-tilt (mmHg)	87 (10)	84 (3)	0.57	0.002	0.05
PetCO_2_ supine (mmHg)	38 (3)	38 (3)	0.85		
PetCO_2_end-tilt (mmHg)	30 (6)	28 (6)	0.57	<0.0001	0.05
CBF supine (mL/min)	615 (71)	632 (71)	0.70		
CBF end-tilt (mL/min)	452 (57)	474 (67)	0.54	<0.0001	<0.0001
CBF end-tilt %change	−26 (5)%	−25 (2)	0.69		
SVI supine (mL/m^2^)	36 (5)	36 (12)	0.95		
SVI end-tilt (mL/m^2^)	25 (4)	23 (8)	0.54	<0.0001	0.01
SVI end tilt %change	−30 (8)%	−35 (7)%	0.27		
CI supine (L/min/m^2^)	2.8 (0.5)	3.2(0.8)	0.17		
CI end tilt (L/min/m^2^)	2.0 (0.3)	2.4 (0.6)	0.08	<0.0001	0.01
CI end tilt %change	−27 (8)%	−26 (6)	0.72		

DBP: diastolic blood pressure; CBF: cerebral blood flow; CI: cardiac index HR: heart rate; norm: normal; DBP: diastolic blood pressure; CBF: cerebral blood flow; CI: cardiac index; HR: heart rate; P_et_CO_2_: end-tidal CO_2_ pressure; SBP: systolic blood pressure; %change: percent change from supine data; SVI: stroke volume index.
